# Videoconference-delivered cognitive behavioral therapy in patients with symptomatic panic disorder following primary pharmacotherapy: a randomized, assessor-blinded, controlled trial

**DOI:** 10.1186/s12888-025-07320-2

**Published:** 2025-09-24

**Authors:** Yoichi Seki, Ryo Takemura, Chihiro Sutoh, Remi Noguchi, Yoko Okamoto, Ikuyo Ohira, Shinobu Nagata, Eiji Shimizu

**Affiliations:** 1 Cognitive Behavioral Therapy Center, Chiba University Hospital, 1-8-1 Inohana, Chuo-ku, Chiba-shi, , 260-8677 Chiba Japan; 2 Clinical and Translational Research Center, Keio University Hospital, 35 Shinanomachi, Sinjuku-ku, Tokyo, 160-8582 Japan; 3 Department of Cognitive Behavioral Physiology, Graduate School of Medicine, Chiba University, 1-8-1 Inohana, Chuo-ku, Chiba-shi, Chiba 260-8677 Japan; 4 Research Center for Child Mental Development, Chiba University, 1-8-1 Inohana, Chuo-ku, Chiba-shi, Chiba 260-8677 Japan

**Keywords:** Antidepressive agent, Anxiolytics, Cognitive behavioral therapy, Panic disorder, Randomized controlled trial, Videoconference

## Abstract

**Background:**

Additional treatment options for pharmacotherapy-refractive patients with panic disorder are needed. Given the difficulty in accessing professional cognitive behavioral therapy in real-world clinical settings, pharmacotherapy remains the typical treatment in patients with panic disorder. However, some patients with panic disorder remain symptomatic despite pharmacotherapy. In this study, to highlight next-step treatment options for patients with panic disorder, we aimed to assess the efficacy of videoconference-based cognitive behavioral therapy for patients with panic disorder who remained symptomatic despite initial pharmacological treatment. To this end, we evaluated the effectiveness of videoconference-based cognitive behavioral therapy for patients as an adjunct to usual care.

**Methods:**

Symptomatic patients with panic disorder following primary pharmacotherapy were randomly assigned to videoconference-based cognitive behavioral therapy or usual care-only groups. The primary outcomes were a reduction in symptomatology assessed at 8 and 16 weeks, using the Panic Disorder Severity Scale. We calculated the 95% confidence intervals (CIs) of the mean with an unknown variance.

**Results:**

Thirty participants were included in this study. After 16 weeks, the adjusted mean changes in Panic Disorder Severity Scale score from baseline were − 7.92 and 0.75 in the videoconference-based cognitive behavioral therapy (*n* = 15) and usual care (*n* = 15) groups, respectively, with a between-group difference of − 8.67 (95% CI: −11.80 to − 5.54; *P* < .0001). A considerable proportion of patients in the videoconference-based cognitive behavioral therapy group achieved a positive response at week 16 (80% vs. 6.7%; *P* < .001) and demonstrated a higher remission rate (66.7% vs. 0.0%; *P* < .001) than that in the usual care group.

**Conclusions:**

Thus, videoconference-based cognitive behavioral therapy is an effective treatment for patients with panic disorder who remain symptomatic following pharmacotherapy. The results suggest that videoconference-based cognitive behavioral therapy, which can be used at home, may be effective for patients with panic disorder or chronic panic disorder who have difficulty accessing cognitive behavioral therapy.

**Trial registration:**

The study was registered in the University Hospital Medical Information Network Clinical Trials Registry (UMIN000029987) on Nov 20, 2017.

**Supplementary Information:**

The online version contains supplementary material available at 10.1186/s12888-025-07320-2.

## Background

Panic disorder (PD) is an anxiety disorder characterized by repeated panic attacks and demonstrates a lifetime prevalence of 3–4% [1]. The diagnostic criteria for PD are defined in the 5th edition of the Diagnostic and Statistical Manual of Mental Disorders (DSM-5) [1] as recurrent and unexpected panic attacks where at least one attack has been followed by at least 1 month of one or both of the following: (1) persistent concern about additional attacks or their consequences; (2) a significant maladaptive change in behavior related to the attacks. Panic attacks, often described as feeling like “dying,” pose a significant threat to patients and have a major impact on their behavior. In this way, they significantly affect daily life [2], as patients with PD may often take time off work or miss school, which may result in unemployment or dropout, and thereby social decline and economic loss. Most patients with PD experience agoraphobia, which affects activities of daily life, as they avoid situations that may lead to a panic attack, that is, situations in which it would be difficult to escape or get help [2]. Hence, PD results in functional impairments (such as social and occupational disabilities), poor health-related quality of life (QoL), and financial burdens [3–5]. Neurotic traits, childhood abuse, and smoking are common risk factors for PD and can be identified several months before a panic attack [2].

PD is often accompanied by other psychological disorders, similar to many anxiety and mood disorders. Importantly, chronic PD is often associated with depression. Approximately 55% of patients with lifetime PD also meet the criteria for lifetime depression, whereas approximately 11% of patients with depression meet the criteria for lifetime PD. Furthermore, coexisting PD and depression could worsen symptoms and result in them becoming long term [6].

Treatment options for PD include psychotherapy and pharmacotherapy [7]. The National Institute for Health Excellence (NICE) guidelines recommend cognitive behavioral therapy (CBT) or antidepressants, including selective serotonin reuptake inhibitors (SSRIs) and tricyclic antidepressants, for moderate-to-severe PD (with or without agoraphobia). CBT is considered the first line of treatment for PD; however, when CBT is not an option, pharmacotherapy can be used. If the disorder is long-standing or the patient’s condition declines or does not respond favorably to psychological intervention, antidepressants are often the treatment of choice [8].

Antidepressants have been found to be more effective than a placebo in the treatment of PD. A recent Cochrane systematic review reported that tricyclic antidepressants outperform placebos in alleviating the symptoms of PD, with no differences between SSRIs and serotonin-norepinephrine reuptake inhibitors [9]. According to the NICE guidelines, benzodiazepines are associated with a suboptimal long-term outcome and should not be prescribed for PD treatment; however, they are still widely used for treating PD, probably due to their faster onset of action [10]. In Japan, benzodiazepines are often prescribed, although no significant improvement was demonstrated in 30–60% of patients with PD who received pharmacotherapy [11]. In most patients with PD refractory to pharmacotherapy, the disorder becomes chronic, but this has yet to be demonstrated in Japanese patients [12].

It is important to explore additional treatment options for patients with PD who remain symptomatic following pharmacotherapy and CBT [13–15]. A meta-analysis of 27 randomized controlled trials (RCTs) reported that CBT is more effective than a placebo in treating anxiety [16]. Studies examining the efficacy of CBT for anxiety-related disorders, including PD, have also found CBT to be a moderately effective treatment [17]. A network meta-analysis on psychological therapies for PD (*n* = 54 trials) [18] demonstrated that CBT was the most extensively studied intervention, often superior to other psychological therapies assessed. The earliest evidence in favor of CBT for PD was reported in the 1990 s, with several representative RCTs showing that it was significantly more effective than standard medications, including alprazolam and imipramine [19–21]. A meta-analysis of 42 RCTs reported that CBT for PD yielded significantly better efficacy outcomes than did pharmacotherapy [22]. A systematic review suggested that psychotherapy alone or psychotherapy plus antidepressants may be selected as the first line of treatment for PD with or without agoraphobia, depending on patient preference [23]. In our previous trial in patients with PD [24], we observed clinically significant reductions in the Panic Disorder Severity Scale (PDSS) scores following an individual CBT program [24–26]. A study by Heldt et al. followed 63 patients with PD who had not responded to previous pharmacotherapy for 1 year after completing group CBT [13]. Although there was no comparison group, approximately two-thirds of the sample met the criteria for remission [13]. In another case study, 12 sessions of group CBT for patients with PD who had failed to respond to 2 months of adequate medical therapy resulted in a significant improvement [14]. Patients with non-remitted PD whose treatment was augmented by face-to-face CBT or medication optimization after 12 weeks of an SSRI showed no between-group differences, suggesting that both interventions are reasonable next-step options [15]. However, the duration of time for defining “PD resistant to pharmacotherapy” was inconsistent across studies, varying between 6 weeks and 4 months. Helds et al., Simon et al., Otto et al., and Kamijima et al. defined drug resistance as patients who did not respond to pharmacotherapy (sertraline or other antidepressants) for 4 months, 6 weeks, 2 months, and 8 weeks, respectively [13–15, 27].

There are also issues regarding access to CBT treatment for patients with PD. Based on estimates from the World Health Organization in 2004, the prevalence of untreated PD was 55.9% [28]. Factors that contribute to patients not regularly attending treatment sessions may include the long travel distance to access mental health services. It may also be difficult for patients with PD and agoraphobia, who often have difficulty leaving the house, to attend weekly face-to-face CBT at outpatient clinics. Therefore, they may prefer pharmacotherapy to face-to-face CBT. Furthermore, CBT is often inaccessible owing to a shortage of therapists and regional imbalances. This is a particularly prominent issue in Japan. Given the difficulty in accessing CBT performed by trained experts in real-world clinical settings, pharmacotherapy remains the typical treatment for patients with PD.

Video conference-based CBT (VCBT) has the potential address the issue of accessibility. VCBT provides access to therapy for patients who are in physically distant locations or unable to leave home owing to severe anxiety or depressive symptoms. A non-RCT of 21 patients with PD showed that 12 VCBT sessions at a remote site were as effective as in-person CBT at the local site in reducing panic frequency, panic apprehension, and agoraphobic cognition [29]. Approximately half the participants had not received treatment for a psychiatric disorder prior to study enrollment. Another study comparing guided and unguided Internet CBT (ICBT) for PD with a waitlist group demonstrated that both treatments were superior to those in the waitlist group, with the guided ICBT intervention outperforming the unguided intervention on most outcome measures [30]. Stubbings et al. [31] conducted an RCT on 26 patients with mood or anxiety disorders, including PD. They found that 12 VCBT sessions were as effective as face-to-face CBT in reducing the symptoms of depression, anxiety, and stress, as well as improving QoL. Finally, a Japanese, single-arm, open study demonstrated that VCBT was effective in controlling and reducing symptoms in patients with PD [32]. Therefore, these findings indicate that VCBT is as effective as face-to-face CBT in patients with PD. However, these studies on VCBT did not include patients with PD who were symptomatic even after undergoing pharmacotherapy.

In this study, we aimed to assess the efficacy of VCBT for patients with PD who remained symptomatic despite initial pharmacological treatment to highlight next-step treatment options for patients with PD. We conducted an RCT to examine the effectiveness of VCBT as an adjunct to usual care (UC) compared with that of UC alone, specifically targeting patients with PD who remained symptomatic following primary pharmacotherapy.

We hypothesized that VCBT would be superior to UC alone in reducing the severity of PD and depressive symptoms and improving the QoL in patients with PD who remain symptomatic following primary pharmacotherapy. If proved effective, VCBT could contribute to increasing treatment options for patients with PD who remain symptomatic after primary pharmacotherapy. This could also help solve the problem of access to CBT treatment for patients with PD.

## Methods

### Study design and participants

This prospective, randomized, open-label, endpoint, single-center study was conducted in the outpatient psychiatry department of Chiba University Hospital in Chiba, Japan, from November 2017 to March 2020. Participants were recruited through posters placed at medical institutions in Chiba prefecture, web advertisements, introductions at patient meetings, and Twitter.

### Inclusion/exclusion criteria

Our eligibility criteria included the following: a primary diagnosis of PD according to the criteria listed in DSM-5 and The Mini-International Neuropsychiatric Interview [33, 34]; aged 18–65 years; PDSS score of at least 8, indicative of active symptomology of moderate severity or higher [35, 36]; having received adequate pharmacotherapy for at least 8 weeks with at least one appropriate dose of an antidepressant, anxiolytic, or other medication that improves the symptoms of PD; or being unable to continue taking medication due to side effects (e.g., nausea, drowsiness, dizziness). When PD was the primary diagnosis, comorbid diagnoses such as depression and generalized anxiety were permitted. The exclusion criteria included psychosis, pervasive developmental disorder or mental retardation, autism spectrum disorders, current high risk of suicide, repeated engagement in antisocial acts, any unstable medical condition, and a lack of informed consent.

All screening interviews were conducted face-to-face by a psychiatrist (ES) and were based on DSM-5 and The Mini-International Neuropsychiatric Interview [33, 34].

We received ethical approval from the Institutional Review Board of Chiba University Hospital (reference number: G29036). All procedures contributing to this work comply with the ethical standards of the relevant national and institutional committees on human experimentation and the Helsinki Declaration. The study was also registered in the University Hospital Medical Information Network Clinical Trials Registry (UMIN000029987; https://www.upload.umin.ac.jp/cgi-open-bin/ctr/ctr_view.cgi?recptno=R000034247.) on Nov 20, 2017. Written informed consent was obtained from all patients after being fully briefed on the procedures prior to screening for eligibility.

### Randomization and masking

After screening, eligible patients were randomly assigned to VCBT or UC groups with a 1:1 ratio using a minimization method based on biased coin allocation balancing with the assignments by the data center at Chiba University Hospital.

There were two allocation modifiers: PDSS severity and sex. PDSS severity was assigned based on the PDSS score cut-off value of 12 based on the average value of 12.1 obtained from the individual data in our previous study [24]. The randomization assignment is the responsibility of the data center at the time of case enrollment; therefore, researchers and clinicians are not informed of the detailed procedure. The structured PDSS evaluations were performed by independent assessors blinded to group allocation without contact with the patients. After PDSS evaluation, the groups were unmasked for guesswork on group allocation with the correct answer rate being recorded.

Participants in the VCBT group received VCBT as an adjunct to UC, and those in the UC group received UC alone. Patients in both groups continued to be treated by their primary care physician. Participants in the VCBT group received VCBT as an adjunct to UC. Participants in the UC group received no other treatment.

### Procedures

#### VCBT

Cisco WebEx (Milpitas, CA, USA) with ISO27001 (regarding handling of information security) and SSAE16 (Statement of Standards for Attestation Engagements No. 16: former SAS 70) compliance certification (issued by a third party), which complies with the United States Health Insurance Portability and Accountability Act, was used. Participants entered a video conference room by clicking a URL in an email sent from their therapists. The VCBT module was based on a previous Japanese study of face-to-face CBT for PD [24] and included psychoeducation, attention shift training, and behavioral experiments. The theme of each session was predetermined, and the same program was implemented for all participants. The VCBT session began with a review of the previous session; homework was assigned after every session to help patients evaluate their beliefs about each collaboratively identified treatment theme in daily life. Homework was sent to patients via email for them to fill out and return. The CBT program in the current study (Additional file 1) was used in our previous studies [24–26] and is based on Clark’s PD model, including themes such as conducting role play-based experiments with and without safety behaviors, reconstruction of catastrophic images from panic attacks into realistic and safe images, and behavioral experiments to test the patient’s negative beliefs [37–39].

This has become the standard CBT program for PD in Japan, and the Ministry of Health, Labor and Welfare has prepared the contents, including explanations, available to the public [40]. The CBT program followed in our current study was based on the one developed and used by our group in a previously conducted single-arm, uncontrolled trial that included 15 patients with PD and revealed clinically significant reductions in the PDSS score from 12.1 at baseline to 5.5 at week 16 [24–26]. We also observed the efficacy of imagery rescripting for early traumatic memories in PD [26]. The CBT program for PD led to significant improvements in mental defeat and cognitive flexibility [25]. There were also case reports of postpartum PD using this program, where anxiety and PDSS scores decreased in all three participants, with two no longer meeting the clinical criteria [41]. In the current study, patients attended VCBT sessions once per week for 16 weeks, with each session lasting 50 min. The therapist and patient used video and audio links to have a therapeutic conversation during VCBT, which included real-time therapist support via videoconference, like face-to-face CBT [32].

#### Pharmacotherapy

Attending physicians continued UC for patients in both groups. UC was defined as continued pharmacotherapy by the primary care physician. The drug dose and type administered as primary pharmacotherapy remained unchanged during the study period, except in unavoidable circumstances. The use of other psychotherapies was not allowed in either group. Any treatment change and the reason for it was recorded throughout the study. During the study period, the researcher had no contact with the attending physician.

#### Therapist and therapy quality control

Individual VCBT sessions were provided by four licensed psychologists experienced in providing CBT to patients with PD. All therapists had completed the Chiba CBT training course, a Japanese adaptation of the UK Improving Access to Psychological Therapies Project [42]. During the Chiba CBT training course, therapist competence was assessed with the Revised Cognitive Therapy Scale [43]. To monitor adherence to treatment and quality control of CBT, all therapists participated in individual weekly supervisory sessions throughout the study.

#### Adverse events

Any adverse events that occurred were reported, regardless of their causal relationship with the study. Events that worsened anxiety, such as panic or depressive symptoms due to discussing past painful experiences, were considered adverse events. The therapist asked the participants about adverse event experiences at each assessment.

### Outcomes

Evaluations were conducted at four time points: screening, week 0 (baseline), week 8 (mid-intervention), and week 16 (post-intervention). The interval between the screening and baseline (week 0) was 2–8 weeks.

#### PDSS

The primary outcome was a change in PDSS score at week 16 from baseline (week 0). The PDSS measured the severity of PD on a 5-point Likert-type scale ranging from 0 (not severe) to 4 (severe); as such, higher scores indicated more severe PD. It is the most frequently used scale for the assessment of PD. The scores were determined by two independent evaluators using a semi-structured interview. Both evaluators had undertaken the same PDSS video training in Japan (scoring while watching simulated interviews of standardized patients) to eliminate inter-assessor bias. In our study, we used the validated Japanese version of the PDSS developed by Yamamoto et al. [36]. Response to treatment was defined as a 40% or greater reduction in the PDSS total score from baseline (week 0) to week 16, based on previous research by Barlow et al. [44]. Remission was defined as a final PDSS score less than 8 points at week 16 [35].

#### Secondary outcome measures

One secondary endpoint was the Panic Agoraphobia Scale (PAS) [45, 46], which is a measure of self-reported PD severity, to confirm whether our results were consistent with those of previous VCBT studies. The PAS comprises 13 items that measure the severity of panic symptoms on a 5-point Likert-type scale. The Japanese version of PAS was developed and validated by Kaiya et al. [46].

Patients were also assessed using the 9-item Patient Health Questionnaire (PHQ-9) [47, 48] and 7-item Generalized Anxiety Disorder Scale (GAD-7) [49, 50]. The PHQ-9 contains nine items assessing severity of depression rated on a 4-point Likert-type scale. The GAD-7 comprises seven items that measure the severity of generalized anxiety disorder on a 4-point Likert-type scale. The Japanese versions of the PHQ-9 and GAD-7 were previously developed and validated [47, 50].

QoL was measured using the EuroQol-5 Dimension-5 levels (EQ-5D-5 L) [51–53]. The EQ-5D-5 L contains five items that assess QoL on a 5-point Likert-type scale ranging from 1 (not severe) to 5 (severe). The Japanese version of the EQ-5D-5 L was developed and validated by Ikeda et al. [52]. The EQ-5D is the most commonly used scale internationally for calculating quality-adjusted life-years, which are often used in cost-utility analyses when choosing health outcomes.

All secondary endpoints were self-assessed through online questionnaires.

### Statistical analyses

The sample size was calculated based on a previous single-arm study, where the difference in PDSS scores from baseline was 6.3 (± 4.2) [21]. In a study of five placebo groups, including CBT [43], the change in PDSS scores was 2.52 (± 3.5). Assuming a difference of 3.78 (± 4.2) standard deviations in the change in PDSS scores between the UC and VCBT groups, with a two-sided significance level of 5%, power requirement of 80%, and 10% dropout rate, we calculated the required number of participants to be 44, with 22 patients in each group.

The distribution and summary statistics of the participants’ background data were calculated for both groups. The 95% confidence interval (CI) was calculated as the CI of the mean with an unknown variance. An unpaired t-test and Wilcoxon’s rank-sum test were used for between-group comparisons of the distributions. For categorical variables, a cross table with the allocation group was presented, as well as the frequency and percentage of categories. Its independence was confirmed using Fisher’s exact probability test.

The primary outcome of change in the PDSS score from baseline to week 16 was analyzed using a generalized linear mixed model, with treatment as the fixed effect and baseline PDSS score (PDSS score 8–12 or ≥ 13) and sex as covariates. Point estimates and their 95% CIs estimated using a generalized linear mixed model were used for dimensional outcomes to determine the means at each assessment point (weeks 8 and 16) and to test the between-group differences at weeks 8 and 16. We assessed group and time as fixed effects, interaction between group and time, allocation adjustment factors (PDSS score at enrollment and sex), PDSS baseline values, and study participants nested in group effects as variable effects. We calculated Cohen’s d, a measure of effect size, as the between-mean difference at week 0 (pre-intervention) and week 16 (post-intervention) divided by the pooled standard deviations. Based on Cohen’s d [54], the effect sizes were categorized as small (0.20–0.49), medium (0.50–0.79), and large (0.80 and above). Analyses of secondary outcomes (PAS, PHQ-9, GAD-7, and EQ-5D-5 L) were performed in the same manner as the primary analysis. For the analysis of treatment responders, a contingency table was constructed for the number of successful and unsuccessful treatments in the VCBT and UC groups, and *P*-values were calculated using Fisher’s exact test to evaluate their independence. Statistical analysis was performed using SAS software version 9.4 (SAS Institute, Cary, NC, USA).

## Results

**Fig. 1:**
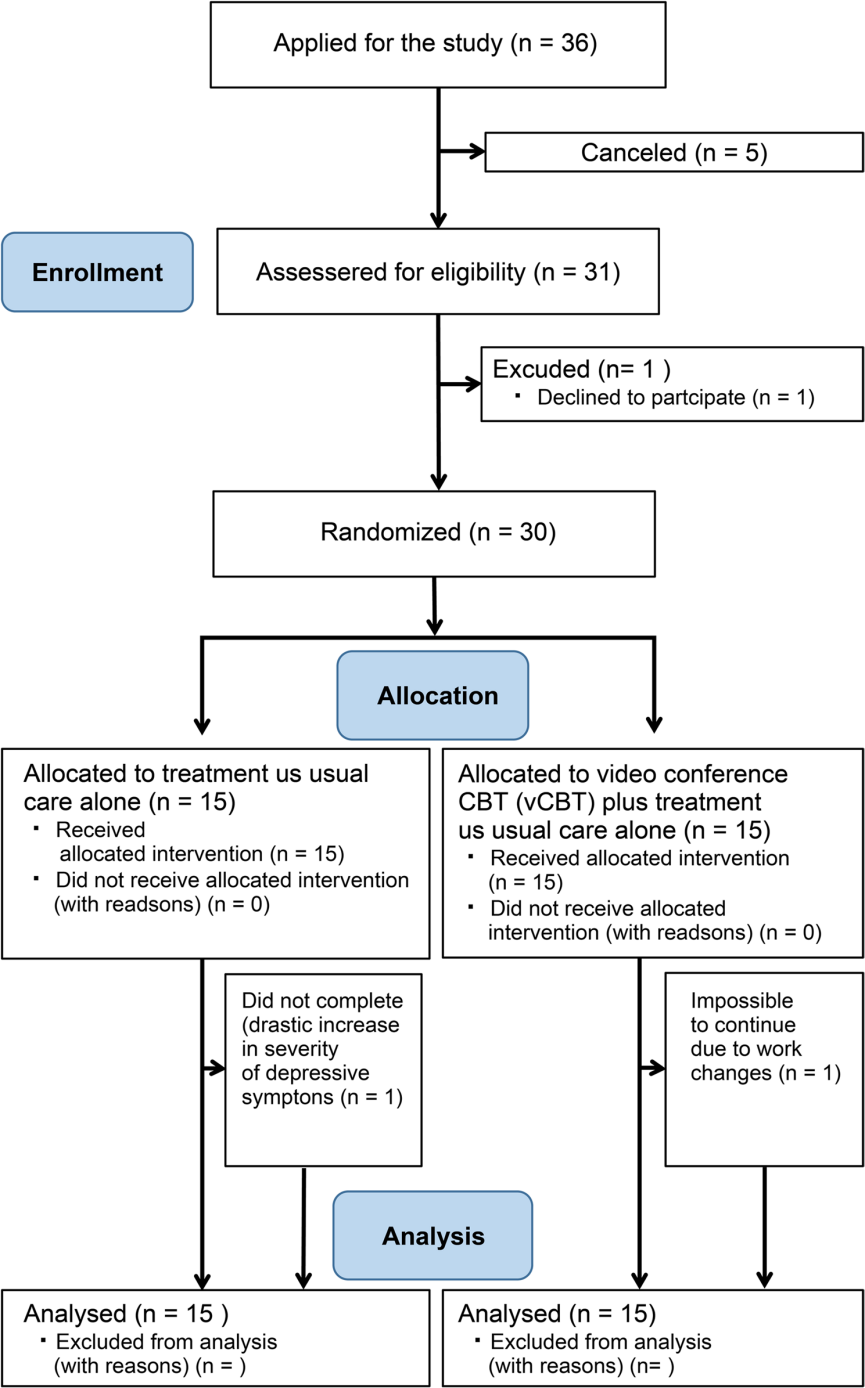
CONSORT flow diagram for the trial. VCBT: videoconference-based cognitive behavioral therapy; UC: usual care

Figure 1 shows the patient flow diagram. Of the 36 patients who applied for participation through our website, 31 underwent face-to-face assessment. One patient later declined to participate; therefore, 30 patients were enrolled in the current study. We stopped recruitment at 30 patients because the recruitment period expired. The original recruitment period was extended for 1 year, but the extended deadline was reached, and recruitment ended with 30 patients.

We randomly assigned 15 patients to each group. In the VCBT group, one patient stopped attending sessions from week 6 for work-related reasons. In the UC group, one patient dropped out of the study after week 1 due to worsening arrhythmia. The data of these patients who dropped out were also included in the intention-to-treat analysis. All patients, excluding one who declined, completed 16 sessions of VCBT. All sessions were conducted per the standardized program shown in Additional file 1. For all participants, the same procedure and program were followed, without deviation. Behavioral experiments were conducted four times as part of sessions 7 to 10.

### Participant characteristics

There were no between-group differences in the participants’ baseline characteristics, as shown in Table 1. All included patients had agoraphobia. Table 1 shows the number of participants in each group who used anxiolytics, antidepressants, antipsychotics, antivertigo drugs, and muscle relaxants. The percentage of benzodiazepines among anxiolytics was 96%. Patients received antidepressants, anxiolytics, or appropriate doses that improved PD symptoms for at least 8 weeks and continued taking the medication until the end of the study, apart from one who was unable to continue taking the medication owing to side effects (severe fatigue).

**Table 1:** Baseline characteristics

Variable		VCBT	UC	intergroup difference
		*n* = 15	*n* = 15	
Male sex		6 (54.5%)	5 (45.5%)	N/S
Age, years		38.4 ± 10.6	40.8 ±7.5	N/S
Years of education		14.4 ± 2.4	14.6 ± 3.4	N/S
Employment status (in paid employment)		8 (53.3%)	12 (80.0%)	N/S
Marital status (married)		10 (41.7%)	14 (58.3%)	N/S
IQ estimated using JART		101.9 ± 7.3	101.5 ± 11.3	N/S
With comorbid metal disorder except for agoraphobia, (All patients had comorbid agoraphobia)		7 (46.7%)	6 (40.0%)	N/S
	Major depressive disorder	1	2	
	GAD	0	1	
	GAD + major depressive disorder	0	1	
	SAD	0	2	
	SAD + GAD	2	0	
	SAD + persistent depressive disorder	1	0	
	OCD	1	0	
	PTSD	1	0	
	Alcohol use disorder	1	0	

Values are presented as n (%) or means ± SD, as appropriate. There were no group differences in any of the variables. This is indicated as N/S

*VCBT* Videoconference-based cognitive behavioral therapy, *UC* Usual care, *IQ* Intelligence quotient, *JART* Japanese Adult Reading Test, *GAD* Generalized anxiety disorder, *SAD* Seasonal affective disorder, *OCD* Obsessive-compulsive disorder, *PTSD* Post-traumatic stress disorder, *PD* Panic disorder

### Primary outcome

**Fig. 2:**
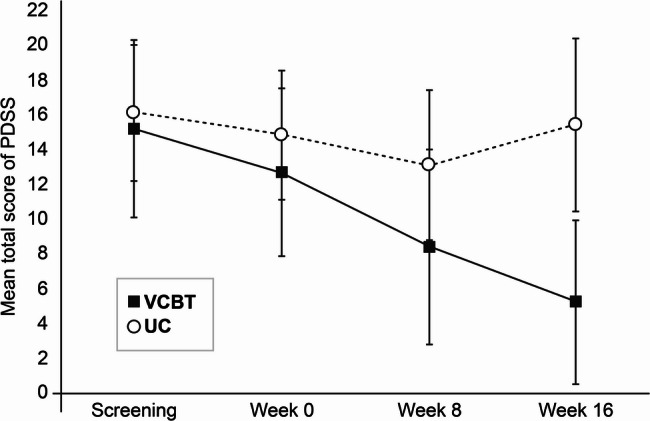
Mean Panic Disorder Severity Scale (PDSS) score at each time point. Error bars represent single standard deviation. VCBT: videoconference-based cognitive behavioral therapy; UC: usual care

Figure 2 shows the mean of PDSS scores across the four time points. Table 2 shows the results of the outcome measurement (see Additional file 2 for the means). At week 16, we observed a significant difference of − 8.67 (95% CI: −11.80 to − 5.54; *P* <.001) in the adjusted mean changes for PDSS scores between the VCBT (− 7.92 [95% CI: −10.14 to − 5.71]) and UC (0.75 [95% CI: −1.47 to 2.96]) groups. Therefore, VCBT significantly improved the PDSS score compared to that with UC, suggesting that VCBT is more effective than UC. In addition, a large effect size (Cohen’s d = 1.19) was observed between the VCBT (1.58) and UC (0.39) groups.

**Table 2:** Adjusted mean changes in PDSS scores

Changes from baseline (week 0)	VCBT (*n* = 15)		UC (*n* = 15)			Intergroup difference		*P *value	
	Least squares mean	95% CI	Least squares mean	95% CI		Difference	95% CI		
PDSS									
Week 8	−4.54	−6.68 to − 2.41	−1.54	−3.75 to 0.67		−3.00	−6.09 to 0.08	0.06	
Week 16	−7.92	−10.14 to − 5.71	0.75	−1.47 to 2.96		−8.67	−11.80 to − 5.54	< 0.001	**
PASX**`**									
Week 8	−4.28	−7.53 to − 1.04	1.31	−1.96 to 4.58		−5.59	−10.21 to − 0.98	0.02	*
Week 16	−9.7.9257	−12.81 to − 6.33	1.31	−1.96 to 4.58		−10.88	−15.49 to − 6.27	< 0.001	**
PHQ-9									
Week 8	−2.33	−4.20 to − 0.47	−0.82	−2.68 to 1.05		−1.52	−4.11 to 1.07	0.24	
Week 16	−2.55	−4.41 to − 0.68	−0.60	−2.46 to 1.26		−1.95	−4.54 to 0.64	0.14	
GAD-7									
Week 8	−1.59	−3.68 to 0.50	−0.07	−2.18 to 2.04		−1.52	−4.5 to 1.46	0.30	
Week 16	−2.88	−4.97 to − 0.79	−0.14	−2.25 to 1.96		−2.74	−5.71 to 0.24	0.07	
EQ-5D-5 L									
Week 8	0.08	0.01 to 0.14	0.02	−0.05 to 0.09		0.05	−0.04 to 0.15	0.26	
Week 16	0.16	0.10 to 0.23	0.04	−0.03 to 0.10		0.13	0.03 to 0.22	0.01	*

Intention-to-treat sample. Measures: higher scores on the EQ-5D-5 L indicate higher quality of life. Higher scores on other measures indicate greater pathology or severity

*PDSS* Panic Disorder Severity Scale, *VCBT* Videoconference-based cognitive behavioral therapy, *UC* Usual care, *PAS* Panic Agoraphobia Scale, *PHQ-9* 9-item Patient Health Questionnaire, *GAD-7* 7-item Generalized Anxiety Disorder Scale, *EQ-5D-5 L* EuroQol-5 Dimension-5 level

*P <.05

**P <.01 Compared with baseline assessment (week 0)

At week 8, the adjusted mean changes in the PDSS score were − 4.54 (95% CI: −6.68 to − 2.41) and − 1.54 (95% CI: −3.75 to 0.67) in the VCBT and UC groups, respectively, with no significant between-group difference (− 3.00 [95% CI: −6.09 to 0.08]; *P* =.06). A significantly higher proportion of patients in the VCBT group achieved a response at week 8 (60%) than that in the UC group (13.3%; *P* =.02), which became more prominent at week 16 (80% response in the VCBT group vs. 6.7% in the UC group; *P* <.001). At week 8, the VCBT group demonstrated a higher proportion of patients who achieved remission (40%) than that in the UC group (26.7%; *P* =.70); however, this result was not statistically significant. Finally, at week 16, a significantly higher proportion of patients in the VCBT group achieved remission than that in the UC group (66.7% vs. 0%; *P* <.001).

### Secondary outcomes

The adjusted mean changes in the PAS score at week 8 were − 4.28 (95% CI: −7.53 to − 1.04) and 1.31 (95% CI: −1.96 to 4.58) in the VCBT and UC groups, respectively, showing a significant between-group difference (− 5.59 [95% CI: −10.21 to − 0.98]; *P* =.02). The corresponding values at week 16 were − 9.57 (95% CI: −12.8 to − 6.33) and 1.31 (95% CI: −1.96 to 4.58) in the VCBT and UC groups, respectively, also showing a significant between-group difference (− 10.88 [95% CI: −15.49 to − 6.27]; *P* <.001; see Additional file 3 for more details).

The adjusted mean changes in the PHQ-9 score at week 16 were − 2.55 (95% CI: −4.41 to − 0.68) and − 0.6 (95% CI: −2.46 to 1.26) in the VCBT and UC groups, respectively; however, these results were not significant between groups (− 1.95 [95% CI: −4.54 to 0.64]; *P* =.14). The adjusted mean changes in GAD-7 score at week 16 were − 2.88 (95% CI: −4.97 to − 0.79) and − 0.14 (95% CI: −2.25 to 1.96) in the VCBT and UC groups, respectively, and were also not statistically significant between groups (− 2.74 [95% CI: −5.71 to 0.24]; *P* =.07). The adjusted mean changes in the EQ-5D-5 L index at week 16 were 0.16 (95% CI: 0.10 to 0.23) and 0.04 (95% CI: −0.03 to 0.10) in the VCBT and UC groups, respectively, with a significant between-group difference (0.13 [95% CI: 0.03 to 0.22]; *P* =.011). Moreover, there were no significant changes in PHQ-9, GAD-7, or EQ-5D-5 L scores from baseline to week 8.

### Quality control

The correct response rate by blind assessors was 53.3% at week 0, 58.6% at week 8, and 78.6% at week 16. At week 16, the assessors commented that they could assume that patients showing improvements belonged to the VCBT group.

### Adverse events

In the VCBT group, one patient experienced two adverse events associated with appendicitis exacerbation. The first exacerbation involved a 6-day hospitalization with drug treatment between weeks 4 and 5, while the second involved a 6-day hospitalization due to surgery between weeks 7 and 8. These events did not occur due to the intervention, and the patient recovered and continued the intervention.

## Discussion

Our study was the first RCT examining the efficacy of VCBT as a next-step treatment for patients with PD who remain symptomatic despite pharmacotherapy. Our findings demonstrated that VCBT was effective as an adjunct to UC in reducing the severity of PD and improving QoL after 16 weeks of intervention.

Previous research has corroborated our findings that combined pharmacotherapy and face-to-face CBT for PD tailored to the primary care setting was superior to usual treatment [55]. In this study, we planned VCBT sessions as the next-step treatment strategy after primary care. This is the first RCT to report that a combination of UC and VCBT is more effective than UC alone, serving as a possible next-step treatment for patients with PD refractory to initial pharmacotherapy in primary care. The effect size of VCBT (Cohen’s d = 1.58) was comparable to that of face-to-face CBT reported in a previous single-arm study (Cohen’s d = 1.77), as well as to the effect size reported in a clinical trial conducted by Barlow et al. [44], wherein the effect sizes (Cohen’s d) were 1.24 for CBT, 1.48 for imipramine, 0.69 for placebo, 1.72 for CBT + imipramine, and 1.41 for CBT + placebo. These results appear promising.

Hence, VCBT and face-to-face CBT may have similar effects for patients with PD. Clarifying the definition of “pharmacotherapy-resistant PD” is impeded by the fact that primary care physicians do not consistently provide guideline-concordant pharmacotherapy in real-world settings. Perna and Caldirola [56] reported that recent studies have not determined the optimal dose/duration of recommended pharmacotherapy before a patient with PD is considered a non-responder. In our study, patients with PD refractory to primary pharmacotherapy were considered those who remained symptomatic (PDSS score of ≥ 8) after receiving initial pharmacotherapy (e.g., antidepressants or anxiolytics) for ≥ 8 weeks or those who discontinued pharmacotherapy due to side effects and were referred to secondary mental health care services. Our findings suggest that VCBT may be useful as a next-step treatment for primary pharmacotherapy-resistant PD.

The change in the PAS score from week 0 to week 16 was only significant for VCBT. Therefore, the effect of VCBT may be an additive effect of the therapy. Similar to the PDSS, the PAS is a measure of panic severity, and significant group differences were found at weeks 8 and 16. Therefore, the results of the PDSS were supplemented.

All patients had comorbid agoraphobia, possibly because the study was conducted online. In a 3-year follow-up study of the chronicity of PD with and without comorbid agoraphobia, 75% of patients with PD experienced remission, compared to only 25% of patients who had PD with agoraphobia, which is more likely to follow a chronic course [57]. The results of PDSS and PAS assessments suggest that VCBT is effective for patients with PD who have comorbid agoraphobia and may also be effective for those with chronic PD.

The results of the PHQ-9 did not show any significant differences between the two groups. The change in the PHQ-9 score from week 0 to week 16 was only significant for VCBT. There was no significant difference in the GAD-7 score between the two groups. However, there was a significant change between week 0 and 16 in the VCBT group. The study did not find sufficient evidence that depression and general anxiety symptoms also improve along with improvement in panic symptoms. These results may be partly due to the low value at week 0 and the sample size. In addition, the low percentage of patients with comorbid depression may have contributed to these findings. In our previous study [24], which used the same program as this study, the changes in PHQ-9 and GAD-7 scores were both statistically significant.

The EQ-5D-5 L assessed QoL and showed a significant difference between the two groups. The change in EQ-5D-5 L scores from week 0 to week 16 was only significantly different in the VCBT group. Therefore, the effect of VCBT for QoL may be an additive effect of VCBT, indicating that QoL improved alongside the improvement in PD symptoms.

Importantly, the definition of pharmacotherapy resistance in PD is unclear. Therefore, future guideline-compliant VCBT studies for PD with a clear definition of pharmacotherapy resistance are needed.

Finally, a comprehensive review of online psychotherapy by Backhaus et al. [58] found no difference in treatment outcomes between face-to-face and online psychotherapy, and satisfaction with the burden of access was higher for online psychotherapy than for face-to-face psychotherapy. PD places a heavy burden on patients in terms of access to medical care and psychotherapy. The results of our study, which aimed to clarify the effectiveness of VCBT for PD, suggest that VCBT can effectively improve treatment access for patients with PD.

### Limitations

This study had some limitations. First, we could not recruit the original planned number of participants. In the initial research plan, the specified recruitment period was set from November 2017 to May 2019. Nonetheless, by May 2019, only 27 participants had been enrolled; therefore, we extended the recruitment period until November 2019. The participant number reached only 30, falling short of the originally planned 44. Unfortunately, we could not further extend the recruitment period, which was subsequently terminated. Nevertheless, a post-hoc power analysis suggested that the sample size was adequate because of the magnitude of the differences between the study groups. Here, the required sample size was originally calculated based on an expected group difference in PDSS scores of 3.78 ± 4.2, resulting in an estimate of 22 patients per group. However, the actual results exhibited a substantially greater difference—8.67— between groups, indicating that sufficient statistical power could have been achieved with a smaller sample size. Thus, the initial sample size calculation likely overestimated the required number of participants. Nevertheless, as this was a single-center study, the results must be interpreted with caution, and larger multicenter studies to confirm our findings are warranted.

Second, since we did not enroll a psychological placebo group, we could not control non-specific factors and target the concrete effects of the VCBT program. Third, the lack of 1-year follow-up data limits the generalizability of our conclusions. Fourth, we did not consider whether the effectiveness of the current CBT would depend on whether the patients in the study had received any psychotherapy, including CBT, in the past. No data were collected to assess this. Finally, the rationale for some exclusion criteria was not clearly described. For example, we excluded individuals who “repeatedly commit antisocial acts,” even if they did not exhibit other dysfunctional or therapy-interfering behaviors. These exclusion criteria were ambiguous and will not be adopted in future studies.

## Conclusions

VCBT is an effective treatment for patients with PD who remain symptomatic after primary pharmacotherapy; additionally, it reduces PD severity and improves the QoL of these patients. The definition of pharmacotherapy resistance in PD remains unclear, and future studies on VCBT consistent with guidelines that clarify the definition of pharmacotherapy resistance are needed.

## Supplementary Information

Additional file 1. Cognitive behavioral therapy program.

Additional file 2. Mean of the patient outcomes.

Additional file 3. Mean Panic and Agoraphobia Scale scores at each timepoint. Error bars represent 1 standard deviation.

## Data Availability

The datasets generated in this study are available from the corresponding author on reasonable request.

## Abbreviations

CBT: Cognitive behavioral therapy
DSM-5: 5th edition of the Diagnostic and Statistical Manual of Mental Disorders
EQ-5D-5L: EuroQol-5 dimension-5 levels
GAD-7: 7-item Generalized Anxiety Disorder Scale
NICE: National Institute for Health Excellence
PAS: Panic Agoraphobia Scale
PD: Panic disorder
PDSS: Panic Disorder Severity Scale
PHQ-9: 9-item Patient Health Questionnaire
QoL: Quality of life
RCT: Randomized-controlled trial
SSRI: Selective serotonin reuptake inhibitor
UC: Usual care
VCBT: Videoconference-based cognitive behavioral therapy
