# Supplementary material for: Videoconference-delivered cognitive behavioral therapy in patients with symptomatic panic disorder following primary pharmacotherapy: a randomized, assessor-blinded, controlled trial
Source: BMC Psychiatry. 2025 Sep 24;25:861. doi: 10.1186/s12888-025-07320-2 (PMC12462340; doi:10.1186/s12888-025-07320-2)
Supplement: Supplementary file 1 — Additional file 1. Cognitive behavioral therapy program. [file 12888_2025_7320_MOESM1_ESM.docx]

**Additional file 2**. Mean of the patient outcomes

| Measures and time points | | VCBT (n=15) |  | UC (n=15) |
| --- | --- | --- | --- | --- |
|  |  | Mean ± SD |  | Mean ± SD |
| **PDSS (range: 0-28)** | |  |  |  |
|  | Screening | 15.3 ± 5.1 |  | 16.2 ± 3.9 |
|  | Week 0 | 12.8 ± 4.8 |  | 14.9 ± 3.7 |
|  | Week 8 | 8.5 ± 5.6 |  | 13.2 ± 4.3 |
|  | Week 16 | 5.3 ± 4.7 |  | 15.5 ± 5.0 |
| **PAS (range: 0-42)** | |  |  |  |
|  | Screening | 24.2 ± 9.0 |  | 25.1 ± 6.6 |
|  | Week 0 | 19.3 ± 9.6 |  | 23.2 ± 5.8 |
|  | Week 8 | 15.3 ± 9.0 |  | 23.3 ± 6.1 |
|  | Week 16 | 10.0 ± 7.0 |  | 23.3 ± 6.7 |
| **PHQ-9 (range: 0-27)** | |  |  |  |
|  | Screening | 10.7 ± 6.3 |  | 11.0 ± 6.2 |
|  | Week 0 | 9.1 ± 5.3 |  | 9.7 ± 5.7 |
|  | Week 8 | 6.9 ± 5.9 |  | 8.9 ± 5.9 |
|  | Week 16 | 6.6 ± 4.5 |  | 9.1 ± 5.8 |
| **GAD-7 (range: 0-21)** | |  |  |  |
|  | Screening | 11.2 ± 6.3 |  | 10.7 ± 5.3 |
|  | Week 0 | 7.5 ± 5.1 |  | 9.7 ± 5.8 |
|  | Week 8 | 6.1 ± 5.3 |  | 8.9 ± 5.0 |
|  | Week 16 | 4.9 ± 4.4 |  | 8.8 ± 4.9 |
| **EQ-5D-5L (range: −0.025 to 1.000)** | | |  |  |
|  | Screening | 0.710 ± 0.2 |  | 0.664 ± 0.2 |
|  | Week 0 | 0.724 ± 0.2 |  | 0.652 ± 0.2 |
|  | Week 8 | 0.793 ± 0.2 |  | 0.712 ± 0.1 |
|  | Week 16 | 0.879 ± 0.2 |  | 0.726 ± 0.2 |

Intention-to-treat sample. Measures: higher EQ-5D scores indicate better QoL. Higher scores on other measurements indicate greater pathology or severity.

VCBT: videoconference-based cognitive behavioral therapy; UC: usual care; PDSS: Panic Disorder Severity Scale; PAS: Panic Agoraphobia Scale; PHQ-9: 9-item Patient Health Questionnaire; GAD-7: 7-item Generalized Anxiety Disorder Scale; EQ-5D-5L: EuroQol-5 dimension-5 levels.
