# Supplementary material for: Videoconference-delivered cognitive behavioral therapy in patients with symptomatic panic disorder following primary pharmacotherapy: a randomized, assessor-blinded, controlled trial
Source: BMC Psychiatry. 2025 Sep 24;25:861. doi: 10.1186/s12888-025-07320-2 (PMC12462340; doi:10.1186/s12888-025-07320-2)
Supplement: Supplementary file 2 — Additional file 2. Mean of the patient outcomes. [file 12888_2025_7320_MOESM2_ESM.docx]

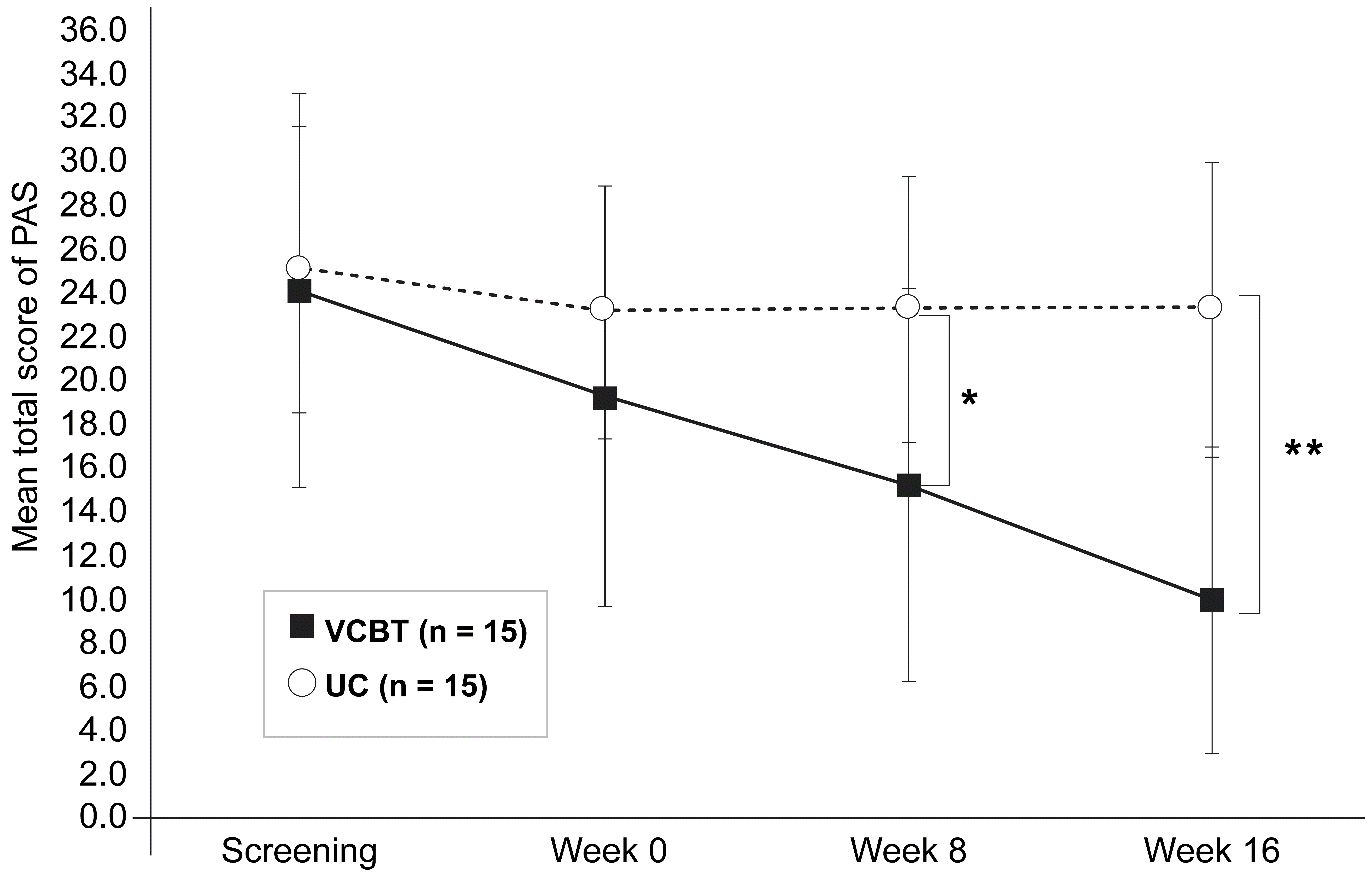


**Additional file 3**. Mean of Panic and Agoraphobia Scale at each timepoint. Error bars represent 1 standard deviation
