# Supplementary material for: Videoconference-delivered cognitive behavioral therapy in patients with symptomatic panic disorder following primary pharmacotherapy: a randomized, assessor-blinded, controlled trial
Source: BMC Psychiatry. 2025 Sep 24;25:861. doi: 10.1186/s12888-025-07320-2 (PMC12462340; doi:10.1186/s12888-025-07320-2)
Supplement: Supplementary file 3 — Additional file 3. Mean Panic and Agoraphobia Scale scores at each timepoint. Error bars represent 1 standard deviation. [file 12888_2025_7320_MOESM3_ESM.docx]

**Additional file 1.** Cognitive behavioral therapy program.

| Session | Program | Description |
| --- | --- | --- |
| Session 1 | Assessment interview | Gain a detailed understanding of the patient's symptoms and medical history, and set treatment goals. |
| Session 2 | Psychoeducation | Provide psychoeducation on panic disorder and cognitive behavioral therapy. |
| Session 3 | Development of an individualized version of the cognitive-behavioral model of PD | Recognize the vicious cycle that exacerbates PD by creating a cognitive-behavioral model for patients. |
| Session 4 | Conducting role-play-based behavioral experiments with and without safety behaviors | Through role-play, patients become aware that their “bias toward safety behaviors and body sensations” in panic situations increases their anxiety. |
| Session 5 | Restructuring catastrophic self-imagery induced by bodily sensations or catastrophic misinterpretations of bodily sensations | Through cognitive restructuring, patients become aware of the difference between their “catastrophic image based on internal information” and their “objectively perceived realistic image.” |
| Session 6 | Practicing external focus and the shifting of attention | Through practice, patients learn to reduce their internal attention to their body sensations and shift their attention in a flexible manner. |
| Sessions 7–10 | Behavioral experiments to test negative catastrophic beliefs (conducted multiple times) | Conduct behavioral experiments on panic situations that involve specific negative predictions made by patients, and obtain actual results to help them realize that the worst-case scenario will not occur. |
| Sessions 11 and 12 | Rescripting early memories linked to negative images in panic situations | Reconstruct the images and past memories that recur during the patient's panic episodes from a new perspective gained through CBT. |
| Session 13 | Modifying problematic pre- and post-event processing | Breaking the vicious cycle of repetitive thoughts and behaviors that occur before and after panic attacks. |
| Session 14 | Discussing the difference between self-beliefs and other people’s beliefs (reflected in survey results) | By actually asking others about the worst-case scenarios and negative predictions that patients fear, they realize that others do not necessarily interpret them in a negative manner. |
| Session 15 | Dealing with the remaining assumptions (schema work) | Enable a flexible perspective on residual beliefs that have been difficult to refute or change in previous sessions. |
| Session 16 | Preventing relapse | The patient reviews previous treatment sessions. |

Note: The same procedure was followed for all participants.
